# Brain Structure and Optimism Bias: A Voxel-Based Morphometry Approach

**DOI:** 10.3390/brainsci12030315

**Published:** 2022-02-26

**Authors:** Raviteja Kotikalapudi, Mihai Dricu, Dominik Andreas Moser, Tatjana Aue

**Affiliations:** Institute for Psychology, University of Bern, Fabrikstrasse 8, 3012 Bern, Switzerland; mihai.dricu@psy.unibe.ch (M.D.); dominik.moser@psy.unibe.ch (D.A.M.)

**Keywords:** optimism bias, voxel-based morphometry, gray-matter volume, putamen, frontal pole, temporal pole, behavior, positive psychology

## Abstract

Individuals often anticipate an unrealistically favorable future for themselves (personal optimism bias) or others (social optimism bias). While such biases are well established, little is known about their neuroanatomy. In this study, participants engaged in a soccer task and estimated the likelihood of successful passes in personal and social scenarios. Voxel-based morphometry revealed that personal optimism bias varied as a positive function of gray matter volume (GMV) in the putamen, frontal pole, hippocampus, temporal pole, inferior temporal gyrus, visual association areas, and mid-superior temporal gyrus. Social optimism bias correlated positively with GMV in the temporoparietal junction and negatively with GMV in the inferior temporal gyrus and pre-supplementary motor areas. Together, these findings suggest that parts of our optimistic outlook are biologically rooted. Moreover, while the two biases looked similar at the behavioral level, they were related to distinct gray matter structures, proposing that their underlying mechanisms are not identical.

## 1. Introduction

Optimism refers to people’s outlook on the world, where they expect more favorable outcomes than unfavorable outcomes [1]. The tendency of adopting a hopeful perspective for the future (without considering specific events) has been termed trait or dispositional optimism and is intimately related to coping behavior [2]. Correspondingly, trait optimism has been suggested to protect against developing depressive symptoms [3,4]. Apart from looking at optimism in more general terms, researchers also investigate optimism bias [5,6,7], namely the degree to (and the conditions under) which we overestimate successes or underestimate risks in well-defined situations. For instance, people believe that good things are more likely to happen to themselves compared to peers and (or) that they will be less likely to experience bad things [5,8]. Such bias can be beneficial toward physical and psychological well-being [9,10,11].

In addition to expressing personal optimism bias (implying greater chances and lower risks for the self with respect to a comparison person or objective standards), people exhibit biased expectancies for social groups (in-groups and out-groups), i.e., social optimism bias [12,13,14]. Specifically, people tend to overestimate the likelihood of positive outcomes for the individuals and social groups that they like or identify with (in-groups) but underestimate the likelihood of positive outcomes for those individuals and social groups they do not value or do not identify with (disliked out-groups). The reverse pattern can be seen for negative outcomes. In fact, individuals are prone to such biases in various social settings, including sports and politics [12,13,15,16,17,18,19].

The literature points to motivational factors being the basis of optimism bias ([20,21]; see [7,22] for overviews). For example, an individual’s desire to experience rewarding future outcomes may trigger optimism bias [22] in order to enhance individual self-esteem. Therefore, the anticipation of self-relevant rewarding outcomes functions as an incentive. In social scenarios, these enhancing effects will be achieved by social identification processes directed toward in-groups and away from out-groups [23,24,25,26,27]. We [22] postulated that these driving forces leading to optimistic biases are intimately linked with “wanting”, as described by Berridge and Kringelbach [28,29] in their research on reward and pleasure. Correspondingly, the human reward system, in particular the striatum, has been hypothesized to be involved in optimistic biases (see [12,30,31,32], for supportive evidence).

Functional magnetic resonance imaging (fMRI) studies have further reported that the rostral ACC (rACC), ventromedial prefrontal cortex (vmPFC), posterior cingulate cortex (PCC), inferior frontal gyrus, middle temporal gyrus, insular cortex, and the amygdala play an important role in optimism bias ([12,14,33,34,35]; see [22] for a theoretical model). These regions may promote optimism bias via their association with emotional processing [36,37,38,39]. While these findings are promising, it remains unclear whether there are also structural brain characteristics that underlie personal and social optimism bias. Morphological characteristics such as gray matter volume (GMV) might in fact support and/or complement the reported neural activity effects. As gray matter consists of cell bodies, they provide the neural substrate for neural responding and the starting and endpoints for interregional communication. Correspondingly, the investigation of structural brain correlates of optimism bias is strongly indicated. Moreover, although previous behavioral results suggest personal and social optimism bias to be of comparable size [40], little is known as to whether the same brain structures underlie both biases. Because social identification processes are more strongly involved in social compared to personal optimism bias, the former is likely to additionally recruit areas involved in social cognition (e.g., temporoparietal junction (TPG) [41,42]). To overcome this lack of knowledge, the current study investigated morphological characteristics underlying both personal and social optimism biases.

With cortical–subcortical volumetric analyses [43,44,45,46], it is possible to identify important structural correlates motivating optimism bias. For volumetric analyses, voxel-based morphometry (VBM) is a highly popular statistical technique facilitating a voxel-level comparison of local concentrations and volumes of mainly the gray matter [43]. The literature concerning brain morphology studies that have probed personal optimism bias (or related concepts) is small. Chowdhury et al., 2014 [47] investigated update bias, i.e., the degree to which we are ready to update expectancies for ourselves into the optimistic rather than pessimistic direction after having received feedback, a phenomenon that thus might underlie or result from personal optimism bias. The authors reported an enhanced update bias in elderly people (compared to their younger counterparts), which positively correlated with GMV in the dorsal anterior cingulate cortex (dACC). Another research group found trait optimism to correlate with the GMV of subcortical structures such as the thalamus (pulvinar) and its extension into the parahippocampal gyrus [48]. Furthermore, a recent study reported putamen density to predict trait optimism [49], while still, another study depicted a positive association between trait optimism and GMV in the orbitofrontal cortex [50].

To date, only one study [51] investigated the commonalities between personal and social optimism bias and associated concepts with brain structure [51,52,53]. Specifically, increased gray matter thickness of the insula and inferior frontal gyrus correlated with stronger scores for personal optimism bias and enhanced pessimistic expectancies (i.e., decreasing social optimism biases) for a disliked out-group. Furthermore, both personal and social optimism biases varied as a positive function of gray matter thickness in the ACC and PCC. However, in this study, personal optimism bias was assessed via a questionnaire, while social optimism biases resulted from experimental manipulations. Such differences in assessment may impact the observation of similarities and differences between the biases. The current experiment, therefore, relied on an investigation of both personal and social optimism biases through an identical experimental manipulation (adapted from [12]).

Previously, sports fans’ strong preference for an American football team influenced their expectations concerning that team’s chances to win or achieve a successful result in the match, thereby establishing robust social optimism biases [12,54,55]. Using the same paradigm in soccer, we have recently demonstrated that personal and social optimism bias are related to resting-state connectivity [40]. Specifically, both biases increased as a function of reduced integration of salience, central executive, and default mode networks, and reduced segregation between default mode and central executive networks. In the present study, we used the same task data to explore the gray matter structural correlates of optimism bias in both personal and social contexts using VBM. Participants in said soccer game task estimated the likelihood of four similarly talented characters making a successful pass to a fellow team player. The displayed characters were (a) a player representing the self; (b) a player representing a rival player who competed for the same team position as the player representing the self; (c) a player of the team the participant was about to join soon (in-group); and (d) a player belonging to the archrival of the team the participant was about to join soon (disliked out-group). This paradigm allowed us to quantify (1) the participants’ bias toward self with respect to their rival, i.e., personal optimism bias, as the difference between the likelihood estimates for self and rival (self-rival), and (2) the participants’ bias for a successful outcome for their team with respect to its rival team, i.e., social optimism bias, as the difference between the likelihood estimates for in-group and out-group (in-group–out-group; secondary effects investigated are displayed in the Supplementary Materials). Positive scores thus suggest the existence of a personal optimism bias or a social optimism bias, respectively. By contrast, negative scores reflect the existence of pessimism biases, and zero scores the absence of bias.

In sum, we aimed to identify the cortical and subcortical gray-matter correlates of variations in two types of optimism, i.e., personal, and social optimism bias, by the application of a VBM approach. Such analyses yield important insights into (a) how deeply these biases are rooted in the brain, (b) their similarities and convergences, and (c) potential routes for their modification. Because we assumed that “wanting” is the driving force for both types of biases, we expected overlapping GMV correlates for the two biases. Specifically, based on earlier (mostly functional) neuroimaging research on optimistic biases (see [7,22], for reviews), we predicted that higher GMV in the human reward system (in particular the striatum and the vmPFC), ACC, PCC, and inferior frontal gyrus would correlate with both personal and social optimism bias. Yet, we also predicted meaningful differences between the biases. For instance, as social optimism bias relies on social identification processes, it was additionally expected that it correlates with areas identified in social cognition (e.g., TPJ [41,42]).

## 2. Methods and Materials

### 2.1. Participants

We recruited 49 healthy (34 female, 15 male) non-soccer-playing and German-language-speaking candidates with an age criterion between 18 and 40 years (mean = 22.87 ± 3.62 years). Soccer-playing individuals were not included to avoid effects of expertise. Recruitment was made through e-mails, flyers, and the local participant pool at the University of Bern, Switzerland. Exclusion criteria included self-reported neurological conditions, psychoactive substance usage, and left-handedness. Participation was compensated with either course credits or 25 Swiss francs (CHF) per hour. Participants gave written informed consent, in accordance with the guidelines of the Declaration of Helsinki [53]. Experimental procedures were approved by the ethics committee of the canton of Bern, Switzerland.

### 2.2. Experimental Paradigm

**Figure 1:**
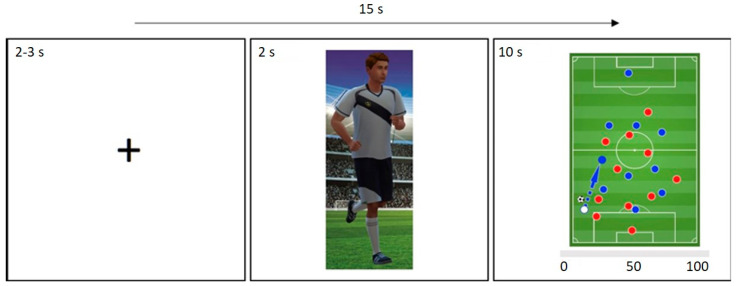
Illustration of an experimental trial. At the start of each trial, a fixation cross was presented for a duration of 2 to 3 s (variation due to jittering). Next, a player representing the self, rival, in-group, or out-group was displayed for 2 s. Subsequently, a soccer game scenario was presented for 10 s; in this latter phase, our participants estimated the likelihood for a successful pass (rating range 0–100%).

The experimental paradigm is shown in Figure 1. 

Participants were told that the task investigated laypersons’ foresighted thinking in soccer contexts of varying difficulty. They were further informed that different players would be shown to decrease boredom. Specifically, each participant saw four different characters of supposedly similar talent relating to (a) their self and (b) a rival concurring for the same position in the participant’s current soccer team, (c) a team they identified with/desired to join shortly (in-group) and (d) its archrival team (out-group). The participants’ task was to estimate the likelihood for a player to successfully pass the ball to his/her fellow team player (with no feedback being provided after the estimate had been given, due to the nonexistence of a ‘correct’ response). Participants first saw a representation of the player in question and were then shown the situation in which said player had to make the pass. At this point, they also estimated the likelihood of the pass being successful.

The task consisted of two blocks (a + b (relevant to personal optimism bias) and c + d (relevant to social optimism bias)) with randomized trials (n = 24) per character. The task scores of the individual participants across trials were averaged for each character, which subsequently provided the possibility to estimate relative biases, i.e., personal optimism bias and social optimism bias. In brief, personal optimism bias refers to the difference in the likelihood estimations of successfully passing the ball to a team player between the self and the rival. That is, personal optimism bias = likelihood estimates for self—likelihood estimates for the rival. Social optimism bias is an extension of personal optimism bias toward a social (teams) scenario. That is, the estimates of likelihood for the in-group toward a desired outcome in comparison to out-group: social optimism bias = likelihood estimates for the in-group minus likelihood estimates for the out-group.

### 2.3. Magnetic Resonance Imaging (MRI) Acquisition

MRI measurements were conducted across the study cohort within a 3T scanner (MAGNETOM Prisma, Siemens, Erlangen, Germany) using a 64-channel head coil, at the Inselspital, University Hospitals Bern, Switzerland. The MRI protocol consisted of a 3D magnetization-prepared rapid gradient-echo (MPRAGE or T1-weighted) sequence with repetition time (TR) = 2300 ms, echo time (TE) = 2.98 ms, inversion time (TI) = 900 ms, flip angle = 9°, and matrix size = 160 × 256 × 256 with an isotropic spatial resolution = 1 mm^3^. Excluding criteria for MRI data acquisition included failed image reconstruction and excessive motion artifacts observed on structural scans (n = 3 participants). The final analyses were conducted on 46 (34 female) participants.

### 2.4. Image Processing

DICOM images from the MRI were converted to NIfTI format using conversion software dcm2niix (https://github.com/rordenlab/dcm2niix/releases, accessed on 25 February 2022 Computational image processing of T1-weighted images was performed using voxel-based morphometry (VBM) [43]. The processes were carried out with SPM12 (https://www.fil.ion.ucl.ac.uk/spm/software/spm12/, accessed on 25 February 2022 ) in a MATLAB 2017b environment (developed by the MathWorks, Inc., Natick, MA, USA) utilizing the cluster service UBELIX (https://ubelix.unibe.ch, accessed on 25 February 2022) from the University of Bern, Switzerland. ‘fsleyes’ from FSL (https://fsl.fmrib.ox.ac.uk/fsl/fslwiki, accessed on 25 February 2022) served as the viewing software for qualitatively assessing the scans and interpreting the results.

### 2.5. Voxel-Based Morphometry

A detailed description of VBM analyses has been intensively covered elsewhere [43,44,56]. In brief, T1-weighted images were segmented into gray matter (GM), white matter (WM), and cerebrospinal fluid (CSF) tissue probability maps using unified segmentation [54], with default bias regularization = 0.0001 and bias cut-off full width at half maximum (FWHM) = 60 mm. Subsequently, GM and WM classification maps were used to create a group-based custom template in the MNI space using diffeomorphic anatomical registration through exponential lie algebra, DARTEL [55]. Later, native GM maps were normalized to the template with an isotropic voxel size of 1 mm^3^. To rectify the regional tissue (native) changes in the normalization process, we modulated the images to preserve the GM tissue volume within each voxel. Finally, normalized GM volume images were smoothed with a (2 2 2) mm FWHM Gaussian kernels, resulting in a nonstationary smoothing of (4.5 4.3 4.5) mm FWHM.

### 2.6. Statistical Analysis

On our behavioral data, we conducted a repeated-measures analysis of variance (ANOVA) with the factors valence (positive connotation (self and in-group), negative connotation (rival and out-group)) and relevance (direct, indirect) on our participants’ averaged likelihood ratings (more information on valence and relevance are provided in the Supplementary Materials). We further calculated a priori contrasts to address personal (self vs. rival) and social (in-group vs. out-group) optimism bias directly. SPM general linear model (GLM) was used for the voxel-based statistical analysis of VBM-derived individual GMV analyses. We used multiple regression as the factorial design specification. Task scores for self, rival, in-group, and out-group were entered as regressors to find the association between optimism biases (as well as valence and relevance; please refer to the Supplementary Materials) and gray matter volume. Contrasts were used to find positive and negative associations for optimism biases namely, personal optimism bias (self–rival) and social optimism bias (in-group–out-group). Additional covariates included age, sex, and total intracranial volume (TIV = GM + WM + CSF). An explicit binary mask was used to confine the findings to the gray-matter region of the brain. The statistical model was estimated, and results were generated at voxel-wise *p* < 0.005 and cluster-level *p* < 0.05 family-wise error correction, P_FWE_. The resulting cluster-level threshold was k = 333 voxels. Reported findings were labeled as increases/decreases of GMV for a given anatomical brain region, using intensity-based masks derived from the Automated Anatomical Labeling (AAL) atlas [56,57].

## 3. Results

### 3.1. Behavioral Data

**Figure 2:**
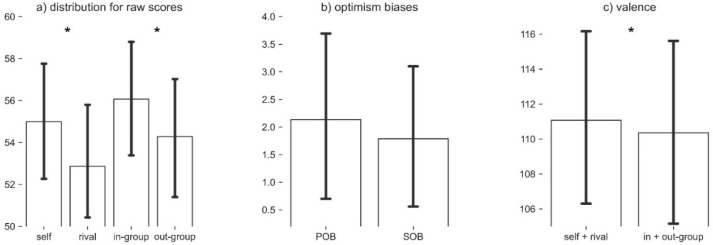
Behavioral data. Behavioral data for the raw scores (**a**) and the derived measures (**b**) are presented. * Indicates a significant difference in the means (*p* < 0.05). Derived measures are POB = self-rival, and SOB = in-group–out-group, where POB stands for personal optimism bias and SOB for social optimism bias. The differences for positive versus negative valence are also shown (**c**).

Behavioral results are shown in Figure 2. 

All the individual likelihood estimate/task scores (self, rival, in-group, and out-group) were within a range of mean ± 3 times the standard deviation. The ANOVA revealed a significant main effect of valence, *F* (1, 45) = 12.46, *p* < 0.001. Positively valenced players (self and in-group; *M* = 55.6%) received overall higher ratings than did negatively valenced players (rival and out-group; *M* = 53.2%). The main effect of relevance, *F* (1, 45) = 1.75, *p* = 0.192, and the interaction valence × relevance, *F* (1, 45) = 0.21, *p* = 0.646, did not reach significance. We also calculated a priori contrasts to address personal (self vs. rival) and social (in-group vs. out-group) optimism bias directly. In line with our expectations, both the contrast performed for personal bias, *t* (45) = 2.97, *p* = 0.002 (one-tailed; *M*_Diff_ = 2.57%), and the one for social bias, *t* (45) = 2.96, *p* = 0.002 (one-tailed; *M*_Diff_ = 2.17%), reached significance, with no difference between the two, *t* (45) = 0.46, *p* = 0.646.

### 3.2. GMV for Optimism Biases

**Figure 3:**
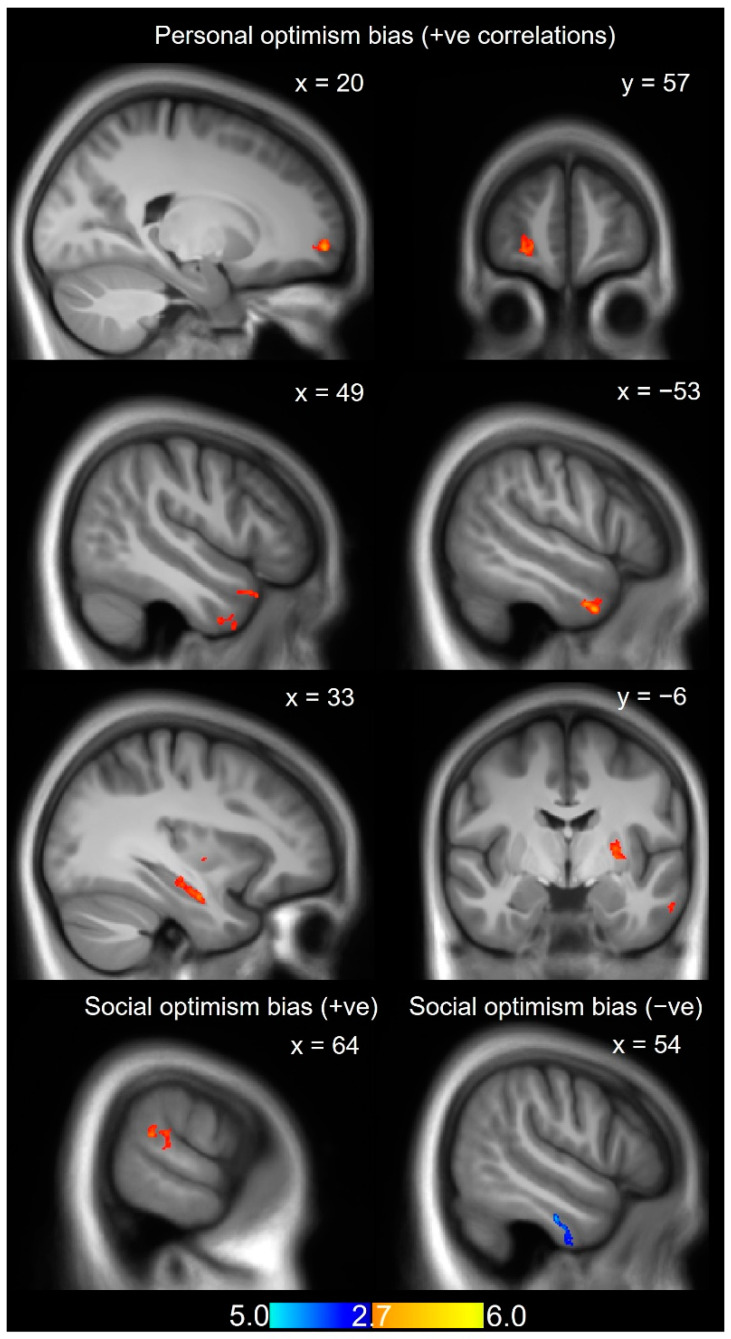
VBM findings for personal optimism bias and social optimism bias. Personal bias: A positive correlation between GMV and personal optimism bias arose in areas including the right frontal pole ((x = 20, sagittal) and (y = 57, coronal)), right temporal pole (x = 49, sagittal), left temporal pole (x = −53, sagittal), left hippocampus (x = −33, sagittal), and left putamen (y = −6, sagittal). Social bias: A positive correlation was observed between GMV and social bias in the right temporoparietal junction (x = 64, sagittal) and a negative correlation in right inferior temporal gyrus (x = 54, sagittal). All findings are overlaid on an average template created by normalized anatomical scans across participants. ‘+ve’ stands for positive correlations between GMV and optimism bias (personal/social), and ‘-ve’ stands for negative correlations between GMV and social optimism bias.

All results for the GMV analysis are presented in Table 1 and Supplementary Tables S1–S3. Personal optimism bias (self vs. rival) positively correlated with GMV in the bilateral temporopolar area, left hippocampus, putamen, and inferior temporal gyrus, as well as right frontopolar region, visual association area, and middle superior temporal gyrus (Table 1, Figure 3). These correlations could be further disentangled: some were driven by positive correlations with the likelihood estimates for the self (left putamen and right frontopolar region), others by negative correlations with the estimates for the rival (bilateral temporopolar regions, right frontopolar region, right visual association area, and the middle superior temporal gyrus, left hippocampus, and left inferior temporal gyrus (Supplementary Tables S1 and S2)). We note that the right frontopolar region was the only structure that correlated both with the self and the rival.

Social optimism bias (in-group vs. out-group) correlated positively with GMV in the right TPJ (Figure 3) and negatively with GMV in the right inferior temporal gyrus (overlapping with a cluster revealing a positive correlation between GMV and likelihood ratings for the out-group) and the left pre-supplementary motor area (overlapping with a cluster revealing a negative correlation between GMV and likelihood ratings for the in-group; Supplementary Tables S1 and S2). Importantly, the respective clusters identified for personal and social optimism bias did not overlap.

## 4. Discussion

In this study, we investigated the cortical and subcortical correlates of personal and social optimism bias. The latter were assessed using a soccer paradigm, in which respondents assessed the likelihood of successfully passing a ball to a team player for (a) oneself, (b) a rival, (c) an in-group player, and (d) an out-group player. Both personal and social optimism bias likely serve to increase personal self-esteem, either directly (comparison of the self with another) or indirectly via social identification processes (comparison of the in-group with the out-group). Correspondingly, the (possibly unconscious) anticipated achievement of a desired end-state (i.e., high self-esteem) was presumed to motivate “wanting” responses [28,29], thereby creating the two biases. In our paradigm, anticipating a more successful pass for oneself compared to a rival player can be a rewarding experience that increases self-esteem. It may hence trigger personal optimism bias (i.e., attributing higher chances of successful passes for oneself than for the rival), in line with motivational accounts of optimism bias [20,21,22] and empirical disclosures (e.g., [12,18]). Similar mechanisms are assumed for social optimism bias.

Due to this assumed shared origin, we predicted their structural correlates to be partly overlapping, particularly in the human reward system (i.e., striatum and vmPFC [22]). Yet, we also expected differences in the GMV correlates for personal and social optimism bias because of partly distinct processes being involved in their genesis. For instance, social optimism biases should substantially rely on social cognition and therefore likely recruit other or additional brain areas.

Whereas our behavioral data suggested that the two biases are comparable (aligning with similarities in resting-state activity [40], we did not observe any overlap between GMV correlates for personal and social optimism bias. This suggests that these biases are differentially rooted in gray matter while sharing some functional aspects, as revealed in fMRI studies. Thus, the two biases may not rely on identical mechanisms.

Consistent with our prediction of human reward activity being at the basis of optimistic biases, we observed a positive association between the size of personal (but not social) optimism bias and GMV in the left putamen. Additionally, we found that personal optimism bias correlated with GMV in bilateral temporal poles, left inferior temporal gyrus, left hippocampus, right visual association area, and the right mid-superior temporal gyrus. By contrast, the increase in social optimism bias correlated, in line with our predictions, positively with GMV in the right TPJ (possibly reflecting increased potential for social cognition; for a detailed discussion, see below) and negatively with GMV in the right inferior temporal gyrus and the left pre-supplementary motor area.

The association between personal optimism bias and the left putamen was driven exclusively by a positive association between GMV and likelihood estimates for the self. This finding is consistent with Sharot et al.’s [30] finding of dopamine being implicated in updating bias, the tendency to more easily change future expectancies into the optimistic rather than pessimistic direction. The putamen, as an integral part of the dorsal striatum, has a causal link to processing rewards. For example, in vivo studies show phasic dopamine release in the putamen in response to food and liquid rewards [58]. Animals are also more receptive to rewards as a function of the number of dopamine receptors in the putamen [59]. Furthermore, single neurons within the putamen can distinguish between minute differences in reward [60]. Finally, the putamen plays a crucial role in informing reward decision making via direct structural and functional pathways to the frontal cortex [61,62,63,64,65,66]. Correspondingly, we suggest that a higher volume of the putamen may predispose individuals toward a larger personal optimism bias via an exacerbated weighing of reward and value. Notably, our findings for the left putamen are in line with a recent VBM study that reported how trait optimism positively correlated with gray matter density in bilateral putamen, with a stronger effect in the left putamen [49]. Furthermore, there is mounting evidence of putamen volumes being negatively associated with depression [67,68,69,70], a condition notorious for its pessimistic expectancies [71,72] and a lack of asymmetrical update of beliefs in the optimistic direction [73].

Consistent with the idea of the human reward system being involved in biased future expectancies, we further observed an association between GMV in the frontal pole and personal optimism bias. The correlation between personal optimism bias and GMV in the right frontal pole was the only one driven by both the estimates for the self and the rival, but in opposite directions: positive correlation for the self and negative for the rival. The right frontal pole is a structure intimately involved in reward-based decision making [74,75,76,77,78]. A frontal pole that is particularly active [74,79,80,81,82,83], stimulated [81,83], larger in volume (VBM [82]), or simply intact [75,84] predisposes individuals to disproportionately weigh prediction errors when choosing which reward to pursue. Occasionally, this leads to a cognitive bias (e.g., because assets that have historically proven to be good value are abandoned [75,85]). Translated to our paradigm while also considering our findings concerning the left putamen, it is tempting to speculate that based on the biased valuation of the self (i.e., left putamen) a larger volume in the right frontal pole further biases the decision to overestimate oneself and underestimate the rival in successfully passing the ball. Future connectivity studies may be able to confirm the potentially concerted action between the putamen and the frontal pole in generating personal optimism bias.

The positive correlations between personal optimism bias and GMV in bilateral anterior temporal poles, left hippocampus, left inferior temporal gyrus, right visual association area, and the right mid-superior temporal gyrus were driven by a negative correlation of GMV with the estimates for the rival, the subtrahend in the personal optimism bias score. This suggests that personal optimism bias in the current study was also substantially driven by downplaying the chances of the rival to successfully pass the ball.

Bilateral anterior temporal poles play a crucial role in encoding and storing both social and nonsocial conceptual knowledge [86,87], as shown by studies on lesion patients [88,89] transcranial magnetic stimulation [88], intracranial recordings [90], and metabolic dysfunctions [91,92]. We speculate that increased volume in the temporal poles makes the respondents more sensitive to the semantic concepts of the self and the rival, leading them to ultimately underestimate the chances for the rival as a method to reduce cognitive dissonance with regard to resource competition (cf. motivational accounts of overoptimistic expectancies such as revealed in wishful thinking, e.g., [20]). Consistent with such an interpretation of the data, our findings also relate to a recent neuroimaging study in which brain activity in the right anterior temporal pole significantly predicted the magnitude of social pessimism bias displayed toward a disliked and denigrated out-group [14].

Social optimism bias (in-group vs. out-group) correlated with GMV in regions distinct from those involved in personal optimism, namely positively, as predicted, with the right TPJ, and negatively with the right inferior temporal gyrus and the left pre-supplementary motor area. The right TPJ is a landmark region involved in various facets of social cognition [93,94,95,96,97,98], including in-group vs. out-group differentiation [99,100]. Although the right TPJ has a much broader role in visual attention and flexibly switching between interoceptive and exteroceptive perception [101], one hypothesis is that evolution may have repurposed voluntary attention and spatial orientation for social cognition [102]. As such, it is not surprising to see the TPJ associated with social optimism bias.

Notably, the findings of the current study do not fully overlap with earlier findings on gray matter correlates of trait optimism [48,49,51] or related concepts [47]. In contrast to other investigations of optimism bias, here we used a competitive context, thereby possibly enabling slightly different mechanisms that rely on additional or distinct brain structures. That we observed differences between the biases here and past literature on trait optimism and updating bias may further be explained by the existence of meaningful differences between these psychological concepts. For instance, trait optimism assesses future expectancies in a very broad, situation-nonspecific manner and does not need to be biased per se. Updating bias, by contrast, investigates the processing of feedback related to initial expectancies (something that we did not provide in the current investigation). Furthermore, divergences between gray matter correlates of the social optimism biases studied earlier [51] and the current study results may relate to methodological differences. For example, the calculation of the optimism biases differed substantially: Whereas in the Moser et al. study [51], an optimism bias was calculated for each character involved (i.e., by subtracting the likelihood estimates for negative outcomes from those for positive outcomes), optimistic biases in the present experiment referred to differences between different characters (i.e., self vs. rival, in-group vs. out-group). This was because our participants’ task was to rate the likelihood of a positive outcome for each player considered (i.e., successful pass), but in no case the likelihood of a negative outcome (i.e., failed pass). Additionally, out-groups in the Moser et al. study [51] were characterized by stereotypical descriptions regarding their warmth and competence. By contrast, the players in the current investigation were not described by any personality characteristics, thereby leaving more space for interpretation to our participants, which may have weakened the effects observed, including potential overlaps between personal and social optimism biases. The exact determinants of the differences in our findings across those studies remain to be determined by future investigations.

Two limitations of our study need to be addressed. First, most of the participants in our study were females (n = 34 out of 46, approx. 74%). To mitigate any potential confounds resulting from such an unequal distribution of the two sexes, we treated total intracranial volume and sex as the covariates in the general linear model. Future studies with a higher sample size could facilitate a systematic comparison of sex differences and optimism bias, which we did not yet find with our study (POB versus sex: point biserial correlation r = 0.04, *p* = 0.80, SOB versus sex: r = −0.09, *p* = 0.55, both tested for 10,000 permutations). Second, our sample size was smaller than several studies with similar methods (e.g., [48]). However, our findings are in line with theoretical models of optimism [14,103] and should serve as a proof of concept that can stimulate further research.

### Implications and Conclusions

Our study is the first to investigate simultaneously the structural correlates of personal and social optimism biases within a comparable experimental setting. We found distinct neural correlates for personal and social optimism bias, suggesting at least partly different mechanisms for generating these biases. Somewhat unsurprisingly, the neural correlates for personal optimism were far more extensive than for social optimism and were driven by overestimating the likelihood estimates for the self and by underestimating the likelihood estimates for the rival. At face value, these findings could be interpreted as optimism bias (especially the personal form) being deeply rooted in the brain and thus hard to overcome or at least be reduced. However, we argue that this very knowledge of brain–behavior associations can be used to develop targeted interventions, such as training participants in a task that taps into the functions of a region of interest, which can lead to significant structural changes in that region. For example, in one study [82] respondents were trained for four weeks in a complex decision-making task and structural changes were observed in the region putatively involved in the type of decision making targeted by the training. Specifically, in the role of a fictional manager, participants had to learn how to balance exploitative choices (reap current financial profits) with exploratory choices (capitalize new product and market opportunities), thus tapping directly into the functions of the right frontal pole [74,75,77], overlapping with the region found by us in correlation with personal optimism bias. The training induced a significantly larger increase in the right frontal pole compared to the control group, as assessed by VBM. Furthermore, there was a significant increase in the fractional anisotropy (i.e., a measure of white matter tracts) of the superior longitudinal fasciculus connecting the frontal cortex with parietal regions [82]. Because personal optimism bias is positively associated with well-being and mental health [9,10,11], its enhancement by such training may be indicated in some cases (e.g., in depression).

## Figures and Tables

**Table 1:** VBM results for personal and social optimism bias.

Contrast	T-max	Peak MNI Coordinate	k	Region (Brodmann Area)	Overlap
POB	4.14	40, 10, −41	499	R Temporal pole (BA38)	50.5% Temporal Inf R 18% Temporal Mid R 9.8% Temporal Pole Mid R
POB	5.20	−53, 4, −34	564	L Temporal pole (BA38)	20.9% Temporal Inf L 50.2% Temporal Mid L 28% Temporal Pole Mid L 0.9% Temporal Pole Sup L
POB	4.47	44, 16, 8	592	R Temporal pole (BA38)	31.9% Temporal Pole Mid R 66.2% Temporal Pole Sup R
POB	4.36	−61, −28, −28	570	L Inferior temporal gyrus (BA20)	86% Temporal Inf L 9.1% Temporal Mid L
POB	5.86	−32, −10, −21	348	L Hippocampus	88.5% Hippocampus L 11.5% Para Hippocampal L
POB	5.40	23, −96, −16	478	R Visual association area (BA18)	11.1% Lingual R 6.7% Occipital Inf R 76.8% Occipital Mid R
POB	4.58	61, −20, −3	363	R Mid-superior temporal gyrus (BA22)	100% Temporal Mid R
POB	4.47	−27, −4, 2	499	L Putamen	17.4% Pallidum L 79.2% Putamen L
POB	5.02	20, 59, −5	491	R Frontal pole (BA10)	100% Frontal Sup 2 R
SOB	4.50	63, −37, 19	345	R Temporoparietal junction (BA22)	27.8% Supra Marginal R 57.7% Temporal Sup R
SOB (negative)	4.84	56, −17, −29	377	R Inferior temporal gyrus (BA20)	79.3% Temporal Inf R
SOB (negative)	5.23	−31, 3, 61	354	L Pre-supplementary motor area (BA6)	42.9% Frontal Mid 2 L 56.5% Frontal Sup 2 L

Gray matter volume correlations with the derived scores of personal optimism bias (POB), and social optimism bias (SOB). A detailed overlap of the cluster finding was determined with the AAL atlas. ‘negative’ represents the negative correlation of behavioral data with gray matter volume. L = left, R = right.

## Data Availability

Under the Swiss guidelines of data protection (Ordinance HFV Art. 5), the datasets generated and analyzed during the current study can be made available from the corresponding author upon reasonable request.

## Supplementary Materials

The following are available online at https://www.mdpi.com/article/10.3390/brainsci12030315/s1, Figure S1: GMV results for valence and relevance, Table S1: Gray matter volume correlations with task scores for self, rival, in-group and out-group, Table S2: VBM cluster overlaps between task scores and derived scores, Table S3: Gray matter volume correlations with the derived scores of valence and relevance.
