# Supplementary material for: Brain Structure and Optimism Bias: A Voxel-Based Morphometry Approach
Source: Brain Sci. 2022 Feb 26;12(3):315. doi: 10.3390/brainsci12030315 (PMC8946158; doi:10.3390/brainsci12030315)
Supplement: Supplementary file 1 [file brainsci-12-00315-s001.zip › brainsci-1560406-supplementary.pdf]

Supplementary data

Brain structure and optimism bias: a voxel-based morphometry approach

Raviteja Kotikalapudi*, Mihai Dricu, Dominik Andreas Moser, Tatjana Aue*

Institute for Psychology, University of Bern, Fabrikstrasse 8, 3012 Bern, Switzerland.

*Corresponding author: Tatjana Aue (tatjana.aue@unibe.ch), Institute for Psychology, University of Bern, Fabrikstrasse 8, 3012 Bern, Switzerland.

Raviteja Kotikalapudi (raviteja.kotikalapudi@gmail.com), Institute for Psychology, University of Bern, Fabrikstrasse 8, 3012 Bern, Switzerland.

Secondary effects investigated

We have further determined (c) a more general valence effect, being a combination of personal and social optimism bias (i.e., 'us [self and in-group] versus them [rival and out-group]') and (d) the influence of relevance (i.e., the degree to which self-relevance or individual performance matters; direct [self and rival] versus indirect [in-group and out-group]). The task scores of the individual participants across trials were averaged for each valence/relevance level, which subsequently provided the possibility to estimate the respective valence and relevance effects (valence = [self + in-group] – [rival + out-group]; relevance = [self + rival] – [in-group + out-group]).

Positive scores for the valence effect indicate that participants were biased in favor of themselves over rivals and their favorite team over others. A positive score thus reflects that successful pass are estimated as more likely for self and ingroup than for disliked or concurring others. By contrast, the relevance component asks whether people treat the scenarios differently according to whether it directly concerns their current goals (i.e., both own performance and the rival's performance touch upon the current goal to keep one's position in the team) or more distant goals (i.e., regarding the performance of the team one is supposed to join and performance of the archrival of this team). Correspondingly, a positive score for relevance bias indicates that personally relevant actors (self/rival) are estimated as more successful than actors that are (only) socially relevant (in-group or out-group).

The two contrasts were used to find positive and negative associations with GMV. Valence ([self + in-group – rival – out-group]) was positively correlated with GMV in the right temporopolar area (overlapping with VBM findings for a negative association between GMV in this area and likelihood rating for the rival; Supplementary Table S3, Supplementary Figure S1), the temporoparietal junction (we did not find a significant overlap with any of the task scores) and the right temporal operculum (partially overlapping with the region being characterized by a negative correlation between GMV and likelihood ratings for the rival). Finally, relevance ([self + rival – in-group – out-group]) negatively correlated with GMV in the right temporal pole (overlapping with the VBM finding of a negative correlation between GMV in this area and likelihood estimates for the rival), left fusiform gyrus (right cuneus).

Figure legends

Supplementary Figure S1. GMV results for valence and relevance.

VBM gray matter volume findings for valence and relevance are presented with a sagittal view (along with slice coordinates), overlayed on a normalized average anatomical T1-weighted brain template. Positive correlations for valence (red-yellow) in right temporal pole (x=44), right temporoparietal junction (x=65) and negative correlations for relevance (blue-light blue) in right cuneus (x=19) and right temporal pole (x=44) are shown here.

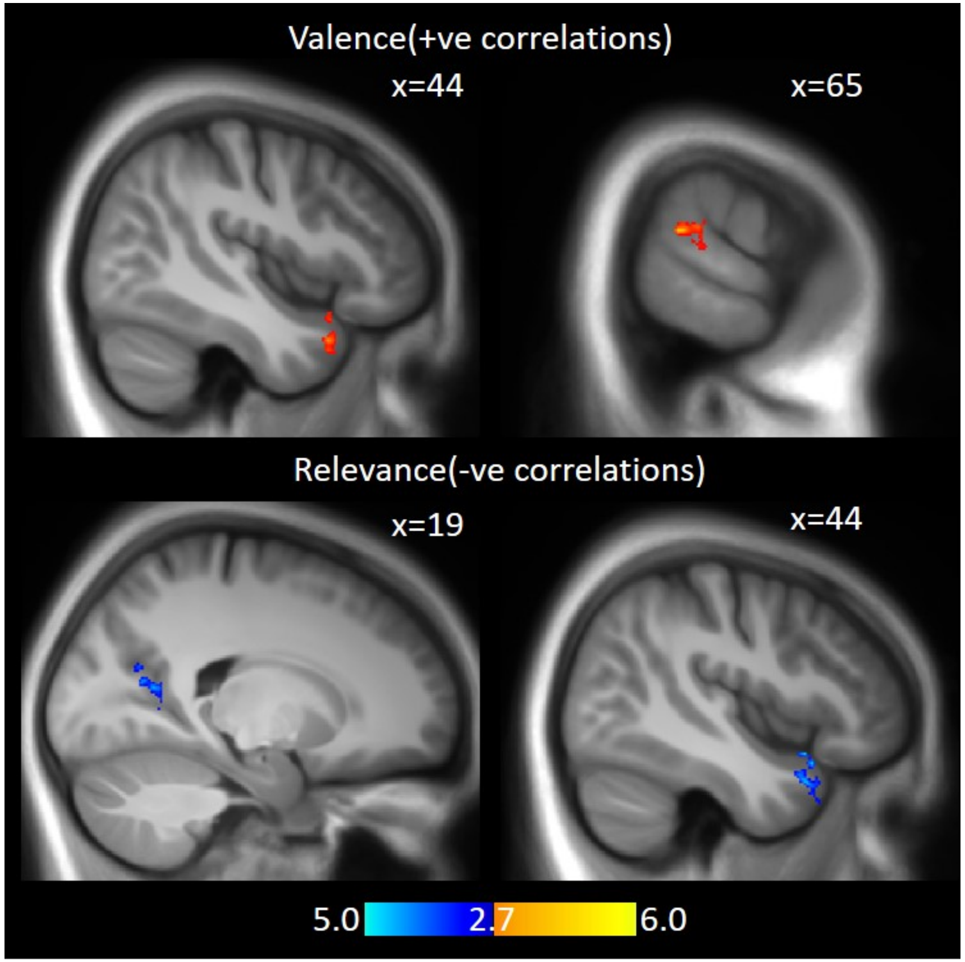

Supplementary Figure S1.

Supplementary Table S1. Gray matter volume correlations with task scores for self, rival, in-group and out-group.

Contrast	T-max	Peak MNI coordinate	k	Region (Brodmann Area)	Overlap
Self	4.56	22, 52,-5	423	R Frontal pole (BA10)	100% Frontal Sup 2 R
Self	4.20	-27, -4, 2	337	L Putamen	13.6% Pallidum L 84% Putamen L
Self	4.57	31,-27, 68	381	R Postcentral gyrus	66.9% Postcentral R 33.1% Precentral R
Rival (negative)	5.28	-53, 4, -34	1115	L Temporal pole (L38)	0.7% Fusiform L 43.6% Temporal Inf L 35.3% Temporal Mid L 20.4% Temporal Pole Mid L
Rival (negative)	4.53	55, 1, -37	679	R Temporal pole (BA20)	36.7% Temporal Inf R 22.2% Temporal Mid R 21.8% Temporal Pole Mid R
Rival (negative)	5.54	-32,-10,-21	899	L Hippocampus	0.1% Amygdala L 15.5% Fusiform L 68.9% Hippocampus L 14.5% ParaHippocampal L
Rival (negative)	5.67	44, 16, -28	1482	R Temporal pole (BA38)	22.5% Temporal Pole Mid R 73.5% Temporal Pole Sup R
Rival (negative)	5.10	-59,-22, -25	764	L Inferior temporal gyrus (BA20)	81.2% Temporal Inf L 13.5% Temporal Mid L
Rival (negative)	5.09	61, -20, -3	1241	R Middle superior temporal gyrus (BA22)	98% Temporal Mid R 2% Temporal Sup R
Rival (negative)	5.26	23,-96, -16	553	R Visual association area (BA18)	7.8% Lingual R 8.3% Occipital Inf R 77.6% Occipital Mid R
Rival (negative)	4.90	20, 60, -5	411	R Frontal pole (BA10)	7.1% Frontal Mid 2 R 92.9% Frontal Sup 2 R
Rival (negative)	3.97	47,-29, 26	750	R Supramarginal gyrus (BA40)	5.1% Postcentral R 21.7% Rolandic Oper R 58.3% SupraMarginal R
In-group	5.12	51, 5, -11	583	R Temporal pole (BA22)	0.2% Temporal Mid R 66% Temporal Pole Sup R 33.4% Temporal Sup R
In-group (negative)	5.01	-37, 6, 55	383	L Pre-supplementary motor area (BA6)	55.9% Frontal Mid 2 L 43.9% Frontal Sup 2 L
Out-group	6.03	55, -17, -28	513	R Inferior temporal gyrus (BA20)	80.7% Temporal Inf R 4.5% Temporal Mid R
Out-group	5.10	-27, -93, -5	338	L Visual association area (BA18)	35.8% Calcarine L 2.1% Lingual L 52.7% Occipital Inf L 5% Occipital Mid L 4.4% Occipital Sup L

A detailed overlap of the cluster findings was determined with the AAL atlas 'Negative' represents the negative correlation of behavioral data with gray matter volume. L = left, R = right.

Supplementary Table S2.VBM cluster overlaps between task scores and derived scores.

	Peak coordinate of correlation with GMV					Peak coordinate of correlation with GMV					Percentage overlap	
Region	Derived score	mm (x)	mm (y)	mm (z)	Cluster size	Task score	mm (x)	mm (y)	mm (z)	Cluster size	Derived score in task score	Task score in derived score
L Putamen	POB	-27	-4	2	499	Self	-27	-4	2	337	66.1	97.9
R Frontal Pole	POB	20	59	-5	491	Self	22	52	-5	423	79.0	91.7
R Frontal pole	POB	20	59	-5	491	Rival	20	60	-5	411	65.6	78.3
R Temporal pole	POB	40	10	-41	499	Rival[1]	55	1	-37	679	77.2	56.7
L Temporal pole	POB	-53	4	-34	564	Rival[1]	-53	4	-34	1115	88.8	44.9
R Temporal pole	POB	44	16	-28	592	Rival[1]	44	16	-28	1482	96.8	38.7
L Inferior temporal gyrus	POB	-61	-28	-28	570	Rival[1]	-59	-22	-25	764	81.1	60.5
L Hippocampus	POB	-32	-10	-21	348	Rival[1]	-32	-10	-21	899	92.0	35.6
R Visual association area	POB	23	-96	-16	478	Rival[1]	23	-96	-16	553	76.4	66.0
R Mid superior temporal gyrus	POB	61	-20	-3	363	Rival[1]	61	-20	-3	1241	90.4	26.4
R Inferior temporal gyrus	SOB[1]	56	-17	-29	377	Out-group	55	-17	-28	513	87.5	64.3
L Pre-supplementary motor area	SOB[1]	-31	3	61	354	In-group[1]	-37	6	55	383	83.9	77.5
R Temporal pole	Valence	44	16	-28	401	Rival[1]	44	16	-28	1482	78.6	21.3
R Temporal operculum	Valence	47	-2	-8	891	Rival[1]	44	16	-28	1482	19.5	11.7
R Temporal pole	Relevance[1]	44	18	-19	1143	Rival[1]	44	16	-28	1482	65.4	50.4

Cluster overlaps between the derived scores (personal optimism bias (POB), social optimism bias (SOB), valence and relevance) and task scores (self, rival, in-group, out-group) are provided along with the MNI coordinates, cluster sizes and percent cluster overlaps. The upper script [1] denoted negative correlation between the contrast and the gray matter volume (GMV).

Supplementary Table S3. Gray matter volume correlations with the derived scores of valence and relevance.

Contrast	T-max	Peak MNI coordinate	k	Region (Brodmann Area)	Overlap
Valence	4.81	44, 16, -28	401	R Temporal pole (BA38)	55.6% Temporal Pole Mid R 43.6% Temporal Pole Sup R
Valence	4.63	47, -2, -8	891	R Temporal operculum	1.3% Temporal Mid R 67.8% Temporal Pole Sup R 30.5% Temporal Sup R
Valence	5.42	65, -39, 19	549	R Temporoparietal junction (BA22)	37.9% Supramarginal R 50.8% Temporal Sup R
Relevance (negative)	4.44	44, 18, -19	1143	R Temporal pole (BA38)	0.1% Frontal Inf Orb 2 R 11.7% Temporal Pole Mid R 84.1% Temporal Pole Sup R 0.1% Temporal Sup R
Relevance (negative)	4.57	-29, -72, -19	627	L Fusiform gyrus	34.9% Cerebellum 6 L 64.4% Fusiform L
Relevance (negative)	4.24	19, -58, 13	412	R Cuneus (BA23)	39.1% Calcarine R 3.2% Cuneus R 4.4% Occipital Sup R 0.2% Precuneus R

A detailed overlap of the cluster finding was determined with the AAL atlas. ‘negative’ represents the negative correlation of behavioral data with gray matter volume. L = left, R = right.
